# Magneto-strain effects in 2D ferromagnetic van der Waal material CrGeTe$_3$

**DOI:** 10.1038/s41598-023-35038-2

**Published:** 2023-05-26

**Authors:** Kritika Vijay, Durga Sankar Vavilapalli, Ashok Arya, S. K. Srivastava, Rashmi Singh, Archna Sagdeo, S. N. Jha, Kranti Kumar, Soma Banik

**Affiliations:** 1 Accelerator Physics and Synchrotrons Utilization Division, Raja Ramanna Centre for Advanced Technology, Indore, 452013 India; 2 Homi Bhabha National Institute, Training School Complex, Anushakti Nagar, Mumbai, 400094 India; 3 Materials Design Division, Department of Physics, Chemistry and Biology (IFM), Linköping University, 581 83 Linköping, Sweden; 4 Glass and Advanced Materials Division, Bhabha Atomic Research Centre, Mumbai, 400085 India; 5 Laser Materials Development and Devices Division, Raja Ramanna Centre for Advanced Technology, Indore, 452013 India; 6 Beamline Development and Application Section, Bhabha Atomic Research Centre, Mumbai, 400085 India; 7 UGC-DAE Consortium for Scientific Research, Khandwa Road, Indore, 452001 India

**Keywords:** Physics, Condensed-matter physics, Electronic properties and materials

## Abstract

The idea of strain based manipulation of spins in magnetic two-dimensional (2D) van der Waal (vdW) materials leads to the development of new generation spintronic devices. Magneto-strain arises in these materials due to the thermal fluctuations and magnetic interactions which influences both the lattice dynamics and the electronic bands. Here, we report the mechanism of magneto-strain effects in a vdW material CrGeTe$_3$ across the ferromagnetic (FM) transition. We find an isostructural transition in CrGeTe$_3$ across the FM ordering with first order type lattice modulation. Larger in-plane lattice contraction than out-of-plane give rise to magnetocrystalline anisotropy. The signature of magneto-strain effects in the electronic structure are shift of the bands away from the Fermi level, band broadening and the twinned bands in the FM phase. We find that the in-plane lattice contraction increases the on-site Coulomb correlation ($U_{eff}$) between Cr atoms resulting in the band shift. Out-of-plane lattice contraction enhances the $d-p$ hybridization between Cr–Ge and Cr–Te atoms which lead to band broadening and strong spin-orbit coupling (SOC) in FM phase. The interplay between $U_{eff}$ and SOC out-of-plane gives rise to the twinned bands associated with the interlayer interactions while the in-plane interactions gives rise to the 2D spin polarized states in the FM phase.

## Introduction

Magneto-strain effect arises due to the intrinsic property of magnetostriction where change in the physical dimension resulted from the magnetic ordering^1^. Magnetostriction couples elastic, electric, magnetic, and thermal fields, and has potential applications in spintronics^2^, straintronics^3,4^, sensors^5^, actuators^6^, transducers^7^ *etc*. In the 2D magnetic materials, there are two different kinds of strain: 1) in-plane strain which is associated with the intralayer coupling and has applications in magnetic switching^8^ and topological switching^9^ and 2) out-of plane strain which is associated with the interlayer coupling and has applications in piezoelectric effects^10^, band gap engineering^11^ and in optoelectronic devices^10,12^.

Recently 2D van der Waal (vdW) materials have attracted much attention in the field of strain engineering due to their extraordinary mechanical properties, like large anisotropic compressibility and Young’s modulus^3,13,14^. CrGeTe$_3$ is a 2D FM vdW material with good thermal stability, ultralow-energy glass formation process, almost zero mass-density change upon crystallization and intrinsic gap tunability^15–17^. These properties of CrGeTe$_3$ have applications in nanoelectronic devices as the magnetic substrate^18^ and next generation memory devices^19^. Bulk CrGeTe$_3$ showed semiconductor behavior with Curie temperature $T_C$ $\approx$ 63 K^18,20,21^. $T_C$ can be tuned with the layer thickness, magnetic field, electric field and strain^15,23–29^. FM ordering exists in atomic layers of CrGeTe$_3$ where Cr atoms are sandwiched between Te atoms and construct a unit of Te–Ge–Cr–Ge–Te with vdW gap between adjacent units^18^. From magnetic studies the moment on Cr atoms is reported to be 2.2–3.0 $\mu _B$ with the out-of-plane easy axis of magnetization and negligible coercivity^15,18,20^.

The FM ordering is reported to renormalize the electronic structure and reduce the band gap which give rise to a slater insulator type behaviour in this system^30^. Experimental band gap ($E_g$) in CrGeTe$_3$ ranges between 0.2 to 0.7 eV^18,30–32^. An indirect $E_g \approx$ 0.38 eV has been determined from angle resolved photoemission (ARPES) measurements by depositing the CrGeTe$_3$ surface with potassium^32^. Density functional theory (DFT) calculation showed that an $U_{eff}$ of about 3–4 eV and exchange energy of 1 eV are required to properly estimate the value of $E_g$ in CrGeTe$_3$^33^. However, the experimental density of states (DOS) suggests that $U_{eff}$ $~\sim$ 1.1 eV is present in this system^31^. ARPES results also showed that the low lying valence band (VB) consists of mainly Te 5*p* orbitals^32^ with multiple hole bands^24^. The single ion anisotropy in this system has been associated with the SOC of Te 5*p* electrons rather than Cr 3*d* electrons^16^. Band splitting and band broadening observed in ARPES spectra below the FM phase transition^34^ have been attributed due to the interplay between localized and itinerant states^23^. Magnetic phase transition from 2D to 3D has been reported due to the small vdW gap and large cleavage energy^35^.

Although there are several studies reported for the magnetic^18,20^ and electronic properties^22,23,31,32,34^ in CrGeTe$_3$ but the microscopic changes for the magneto-strain effects has not been well explored. In this article, we have clearly explained the mechanism of magneto-strain effects in this system from both the theoretical density functional calculations and the experimental evidences. We find that the magneto-strain effect is associated with the isostructural transition in CrGeTe$_3$ across $T_C$ with substantial volume collapse and first order nature. Signatures of magneto-strain effects observed in the electronic structure are shift in the bands away from Fermi level ($E_F$), band broadening and twinned bands in the FM phase. The in-plane lattice contraction gives rise to the increased electron-electron correlation $U_{eff}$ between the Cr–Cr atoms and shifts the bands away from $E_F$. SOC defines the spin-axis in the out-of-plane direction. The out-of-plane lattice contraction give rise to the $d-p$ hybridization between Cr–Ge and Cr–Te atoms which results in band broadening and spin polarization, respectively. As Cr atoms are not arranged one above the other in different layers, hence the increased interlayer interaction out-of-plane gives rise to twinned electronic bands in the FM phase. We have shown that the 2D ferromagnetism, out-of-plane spin polarization and the in-plane magnetocrystalline anisotropy in this system is due to the interplay between the $U_{eff}$ and SOC.

## Results and discussions

**Figure 1:**
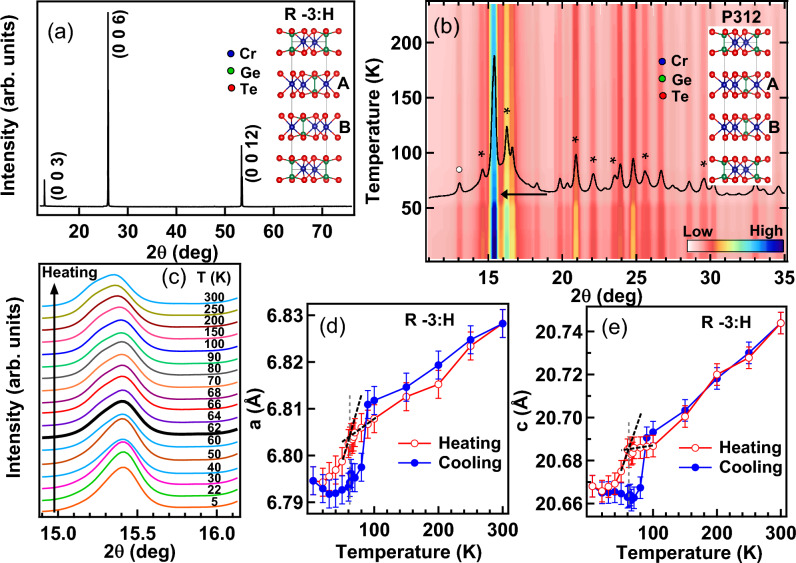
The XRD pattern of CrGeTe$_3$ single crystal recorded using Cu K$_\alpha$ source is shown in (**a**). R-3:H crystal structure is shown in the inset of (**a**). Temperature dependent synchrotron XRD of the CrGeTe$_3$ powder sample in the heating cycle recorded at 15 keV is shown in the contour plot in (**b**). The XRD profile of the powder CrGeTe$_3$ recorded at $T_C \approx$ 62 K (marked by arrow in (**b**)) is superimposed on the contour plot. Asterisk mark ($*$) and open circle ($\circ$) indicates phase segregated lower symmetry P312 phase of CrGeTe$_3$ and the eutectic GeTe phase, respectively. The P312 crystal structure is shown in the inset of (**b**). Splitting of the 15.35 deg peak in (**b**) across the magnetic transition is shown in zoomed scale in (**c**). The variation of the lattice parameters *a* and *c* as a function of temperatures for R-3:H phase in both the heating and cooling cycles are shown in (**d**) and (**e**), respectively. $T_C$ has been determined from the inflection point in the heating cycle (vertical dashed line) in (**d**) and (**e**). The XRD pattern at $T_C$ is shown by black thick line in (**c**).

The X-ray diffraction (XRD) pattern of CrGeTe$_3$ single crystal recorded with Cu K$_\alpha$ source shows (0 0 3n) reflections in Fig. 1a indicating the crystal orientation along the c-axis. All the peaks in the XRD pattern can be indexed with centrosymmetric trigonal R-3:H (148) space group (inset of Fig. 1a) with no sign of impurity phases. The lattice parameters determined from Le Bail fitting : $a=$ 6.8576 $\text{\AA }$ and $c=$ 20.5979 $\text{\AA }$ (see supplementary information) are found to be slightly higher than the reported lattice parameters of CrGeTe$_3$ single crystal ($a=$ 6.8275 $\text{\AA }$ and $c=$ 20.5619 $\text{\AA }$) in the literature^20,36^. The lattice parameter may increase due to small composition variation or intrinsic disorders present. Actual composition determined from EDAX is Cr$_{1.05}$Ge$_{0.83}$Te$_{3.12}$ which is very near to the intended composition. Powder XRD as a function of temperature in the heating cycle recorded with synchrotron source at 15 keV is shown in Fig. 1b. On grinding the single crystal to fine powder for about 2 hours in mortar and pestle, we find prominent coexistence of crystalline and amorphous phases in the synchrotron XRD pattern. Pressure induced phase change by grinding with mortar pestle has been well reported in the literature^37,38^ for the ceramic samples. The grinding is reported to induce about 1–2 GPa pressure which can give rise to the metastable phases Ref.^38^. Presence of metastable crystalline and amorphous phases with the similar local structure has been reported in CrGeTe$_3$^19^. In Fig. 1b we can clearly see that the crystalline intense peak around 15.35 deg is superimposed on an amorphous like broad hump. The amorphization in CrGeTe$_3$ can happen due to the induced strain while grinding. CrGeTe$_3$ is a well known phase change material and pressure induced amorphization in this system has been reported to give rise to short range clusters^16,39^.

Pressure can either introduce crystalline to crystalline polymorphic transition where reconstruction happens in the atomic layer or crystalline to amorphous transition where atoms randomly flip into vdW gaps^39^. We find that the dominating crystalline phase is the centrosymmetric R-3:H phase of CrGeTe$_3$ in Fig. 1b but there are also very small contributions from other phases. These phases are mainly the non centrosymmetric type trigonal unit cell of CrGeTe$_3$ phase with P312 (149) space group^20^ (marked by asterisk in Fig. 1b) and the eutectic GeTe phase with R-3m:H (166) space group^40^(marked by open circle in Fig. 1b). Le Bail fitting of all the phases are shown in the supplementary information. The difference between R-3:H and P312 crystal structure of CrGeTe$_3$ can be clearly seen in the *A* and *B* layers in the inset of Figs.  1a,b where the position of Cr and Ge atoms are interchanged in the CrGeTe$_3$ lattice. Presence of low symmetry P312 structure along with the R-3:H structure is also reported in the powder X-ray diffraction of CrGeTe$_3$ in the Ref.^20^. In addition the Ge and Te centered clusters in the amorphous phase have been predicted from the DFT calculations^16^. The phase change mechanism in CrGeTe$_3$ is governed by the change in the bonding configuration between Cr, Ge and Te atoms^19^. Replacing Cr atoms by Ge atoms prefer to form a Ge-Ge dimer, and causes distortion of Te atoms to achieve the lowest energy and the most stable structure in CrGeTe$_3$^41^. Coexisting phases with pressure or strain originates in vdW material because the layered structure makes the crystal plane slip easily^41^.

We find that there is sudden change in the intensity of the XRD profile below the magnetic transition around 62 K (marked by an arrow in Fig. 1b). The R-3:H peak at 15.35 deg in Fig. 1c shows splitting above 62 K (XRD profile shown in bold black line). Similar behaviour has been associated with the magnetic ordering in many rare-earth based systems^42,43^. The lattice parameters determined using the Le Bail refinement for R-3:H phase in Figs. 1d,e shows a first order type hysteresis around the magnetic transition in both heating and cooling data. We find that there is $\approx$0.6$\%$ decrease in *a* while *c* decreases $\approx$0.4$\%$ across the paramagnetic (PM) to FM phase transition indicating larger contraction along a plane. The lattice parameter variation of P312 phase of CrGeTe$_3$ and GeTe phase are shown in the supplementary information. Both the splitting in the 15.35 deg peak (R-3:H) above and below the magnetic transition and hysteresis in the lattice parameters around the magnetic transition is associated with the magneto-strain effect and have been observed in other magnetic systems like CeAg$_2$Ge$_2$^42^, PrGe^43^ with itinerant magnetism. We have determined the magneto-strain $\epsilon$ for the R-3:H phase using the standard procedure as described in our Ref.^42^. Estimated magneto-strain in CrGeTe$_3$ at 300 K is $\epsilon =~14.313~\times ~10^{-2}$ which is found to increases in the FM phase to $\epsilon =~14.378~\times ~10^{-2}$ at 64 K and $\epsilon =~14.413~\times ~10^{-2}$ at 5 K (detail magneto-strain plots are shown in supplementary information). Hence, the magneto-strain effects in FM phase give rise to a compressive strain ($\approx$ 0.7$\%$ between PM and FM phase) and a larger lattice contraction observed along *a* plane which results in increased in-plane interaction between the Cr–Cr atoms in CrGeTe$_3$. Magneto-strain in vdW materials is reported to vary the vdW gaps^33^ and leads to novel electronic structure and transport properties in these 2D materials which is important for the spintronic applications.

**Figure 2:**
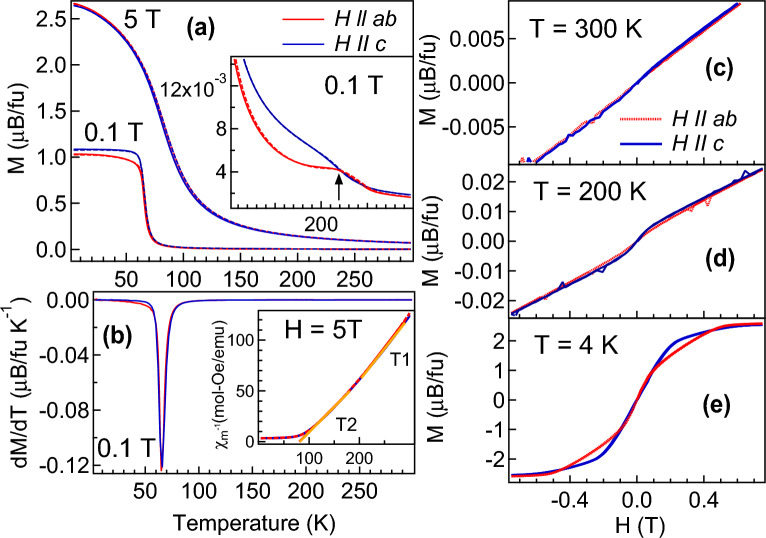
Temperature dependent magnetization at 0.1 T and 5 T field at H//ab plane and H//c axis is shown in (**a**). Zoomed region between 105 to 300 K of M versus T data at 0.1 T is shown in the inset of (**a**) for the signature of short range ordering in H//ab plane and H//c axis. dM/dT versus T plots at 0.1 T field in H//ab plane and H//c axis are shown in (**b**) for the estimation of $T_C$. $\chi _m^{-1}$ versus T at 5 T field is shown in the inset of (b) with the solid lines at T1 and T2 are the fitting using Curie Weiss law. M versus H at 300 K, 200 K and 4 K are shown in (**c**), (**d**) and (**e**), respectively along H//ab plane and H//c axis.

To understand the magnetic interactions in the CrGeTe$_3$ single crystal we have performed the temperature dependent magnetization (M) at 0.1 T and 5 T magnetic field in zero-field-cooled (ZFC) and field-cooled-cooling (FCC) protocol as shown in Fig. 2a with solid and dashed lines, respectively. The magnetization is measured by applying a magnetic field (H) parallel along ab plane (in-plane) and along c-axis (out-of-plane). A clear PM to FM phase transition is observed at both 0.1 T and 5 T in Fig. 2a with higher magnetization along c-axis $\sim$1.076 $\mu _B$/fu than the ab plane $\sim$1.025 $\mu _B$/fu (see 0.1 T data below 70 K) indicating that the c-axis is the easy axis of magnetization. At the 5 T field, the difference in magnetization along ab plane and c-axis is hardly visible in Fig. 2a. The accurate Curie temperature $T_C$ $\approx$ 66 K has been determined at 0.1 T from dM/dT versus T curve as shown in Fig. 2b. We find that the magnetic transition at 5 T is quite broad (Fig. 2a) which clearly indicates the generation of strain with the increase in the magnetic field^44^. It is reported that the magnetic moment on Cr atoms increases monotonically with the tensile strain^44,45^ which has been clearly observed in the present studies (see Fig. 2a). We have also observed a very small signature of a short range ordering around 220 K at 0.1 T (see inset of Fig. 2a) which shows a different behavior along ab plane and c axis and almost negligible at 5 T (see supplementary information). Presence of magnetic correlation around $\sim$160 K in CrGeTe$_3$ is also reported in Ref.^46^ and has been attributed due to either the island of local FM or antiferromagnetic order in a PM host. Short range ordering in the CrGeTe$_3$ has been associated with the self-organizational tendency for atomic rearrangement that derives from the inherent 2D nature and depends on the layer thickness and composition^47,48^. Later in the band structure studies we have shown that this short range ordering is associated with polaronic states localised on the surface and may have arisen due to the bulk surface interaction^49^. The short range ordering has FM correlations which is evident in the M versus H data in Fig. 2d. Small FM correlation along the c-axis is also observed in the PM phase in Fig. 2c which indicates strong exchange interaction in this direction. The molar magnetic susceptibility ($\chi _m$) has been estimated from the value of M and the H at 5 T using the formula: $\chi _m=$ M/H. The effective magnetic moment has been determined by fitting with Curie-Weiss law $\chi _m =$ C/(T-$\theta$) where C is the Curie-Weiss constant and $\theta$ is the Curie-Weiss temperature. In the inset of Fig. 2b, $\chi _m^{-1}$ versus T plot is shown. Due to different slope across short range ordering, $\chi _m^{-1}$ has been fitted in two different temperature range from 205 to 290 K (denoted as T1) and 105 to 180 K (denoted as T2) above and below the short range ordering as shown in the inset of Fig. 2b. At T1, the estimated value of $C_{ab}$($C_c$) $\approx$ 1.598 ± 0.003 (1.559 ± 0.005) emu K/mol and $\theta _{ab}$($\theta _{c}$) $\approx$  103.9 ± 0.3(105.5 ± 0.5) K. At T2, the estimated value of $C_{ab}$($C_c$) $\approx$ 2.001 ± 0.001 (2.011 ± 0.01) emu K/mol and $\theta _{ab}$($\theta _{c}$) $\approx$  81.55 ± 0.3(80.68 ± 0.5) K. The constant $C_{ab}$ and $C_c$ at T1 and T2 gives the information about the magnitude of the moments. We find that the magnitude of moment increases along c-axis (out-of-plane) than ab plane (in-plane) which can be related to the direction of spin-polarization explained in detail later. Higher positive value of $\theta$ than $T_C$ indicates strong FM interactions. The effective magnetic moment defined as $\mu _{eff}$ = p$_{eff}$ $\mu _B$^50^, where $p_{eff}$ = $\sqrt{8C}$, comes out to be 4 ± 0.01 $\mu _B$ per Cr ion at T2 and 3.55 ± 0.03 $\mu _B$ per Cr ion at T1 in both the ab plane and the c-axis. The expected value of Cr$^{3+}$ moment is 3.87 $\mu _B$, however higher value of $\mu _{eff}$ at T2 is due to the magnetocrystalline anisotropy in the FM phase associated with the SOC. Hence, the magneto-strain effects give rise to more magnetocrystalline anisotropy in-plane (ab plane) than out-of-plane (c-axis) as observed in Fig. 2e. The coercivity is found to be negligible in both the directions (in Fig. 2e) indicates that this material is a soft magnetic material.

**Figure 3:**
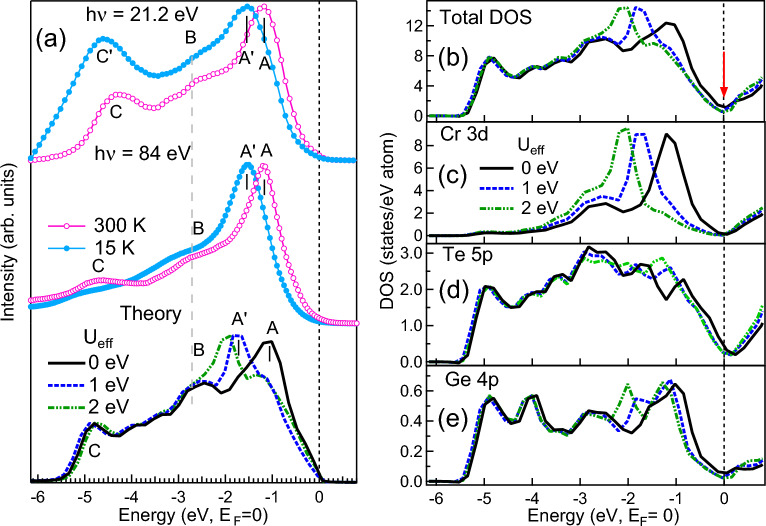
VB spectra of CrGeTe$_3$ (**a**) at 300 K (RT) and 15 K (LT) recorded using 21.2 eV and 84 eV excitation energy and compared with the theoretical VB calculated using GGA + *U* method with $U_{eff}=0$, 1 and 2 eV. (**b**–**e**) show the GGA + *U* calculation for total DOS, Cr 3*d*, Te 5*p* and Ge 4*p* states, respectively with $U_{eff}=0$, 1 and 2 eV. The red arrow shows the position of the conduction band edge in total DOS.

To understand the character of valence electronic states in CrGeTe$_3$ we have measured the VB at 21.2 eV and 84 eV as shown in Fig. 3a and compared with the calculated VB from the DFT calculations. VB has the dominating contribution from the Cr 3*d*, Ge 4*p* and Te 5*p* states, hence the partial density of these states are shown in Fig. 3c–e. Total DOS in Fig. 3b is obtained by adding all the PDOSs after multiplying with the photoionization cross-section at 21.2 eV. The calculated VB as shown in Fig. 3a has been broadened using the standard procedure reported elsewhere^42,43,50,51^. To account for Coulomb correlation effects in the open atomic-like Cr 3*d* orbital, we have performed the GGA $+$ U calculations, where $U_{eff}$ in the Cr 3*d* shell was set to 0, 1 and 2 eV. The total DOS and PDOSs calculated with different $U_{eff}$ are shown in Fig. 3b–e. Experimental VB at 300 K (RT) showed prominent 3 features at -1.19 eV, -2.73 eV and -4.34 eV marked as *A*, *B* and *C* respectively in Fig. 3a which are in good agreement with the calculated VB features for $U_{eff} = 0$. The features *A* and *C* show a shift towards higher binding energy (BE) at 15 K (LT) and marked as $A'$ and $C'$. The experimental VB at LT shows a good matching with the calculated VB for $U_{eff} =1$ indicating that the electron–electron correlation enhances at LT. Similar Coulomb interaction between the Cr 3*d* electrons with $U_{eff} \sim$1.1 eV is reported in Ref.^31^. The conduction band edge is marked by red arrow in Fig. 3b. There is prominent shift in the position of the valence band edge with $U_{eff}$ in the calculation. Larger contribution of Cr 3*d* states is seen in the experimental VB in Fig. 3a due to the higher photoionization cross-section at both 21.2 eV and 84 eV which agrees well with the Cr 3*d* PDOS in Fig. 3c. The feature *A* is the dominating Cr 3*d* state while feature *B* and *C* have dominating Ge 4*p* and Te 5*p* states. The contribution due to Ge 4*p* and Te 5*p* states are about 40 times higher in 21.2 eV than in 84 eV^52^ that leads to larger intensity of these states in Fig. 3a for the 21.2 eV spectra which corroborates with the PDOSs shown in Figs. 3d,e. Moreover, the enhanced intensity of feature *B* and *C* at LT indicates that the $U_{eff}$ between the Cr atoms largely affect the Ge and Te atoms. Higher density of Te 5*p* state at $E_F$ (Fig. 3d) indicates that these states carry the spin polarization.

**Figure 4:**
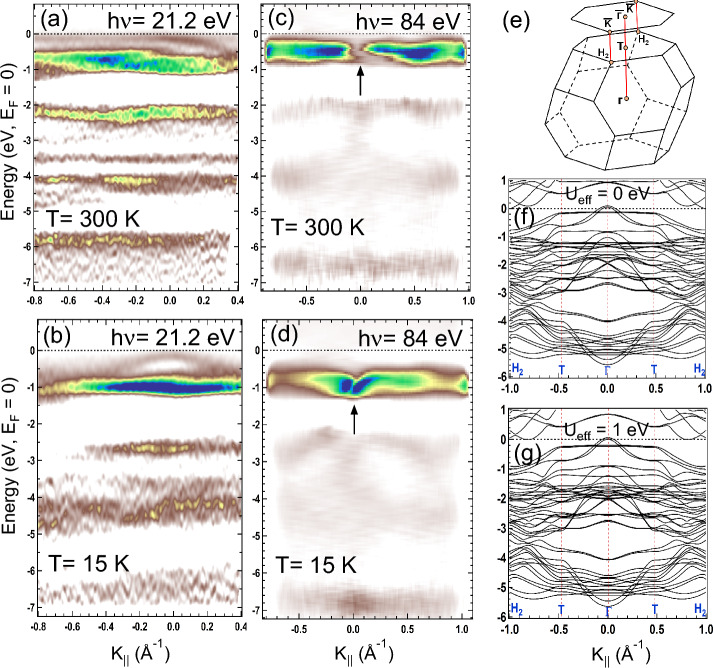
ARPES data of CrGeTe$_3$ measured using (**a**) 21.2 eV at 300 K (RT), (**b**) 21.2 eV at 15 K (LT), (**c**) 84 eV at 300 K (RT) and (**d**) 84 eV at 15 K (LT). (**e**) The Brillouin zone showing the direction $H_2-T-\Gamma -T-H_2$ (in bulk) and ${\bar{K}}-{\bar{\Gamma }}-{\bar{K}}$ (on the surface) where the E-k data are collected and shown in (**a**)–(**d**). Band structure calculation using GGA $+$ U with $U_{eff}= 0$ and 1 are shown in (**f**) and (**g**), respectively.

ARPES band mapping in Fig. 4a–d performed using 21.2 eV and 84 eV photon energies along $H_2-T-\Gamma -T-H_2$ direction in bulk (${\bar{K}}-{\bar{\Gamma }}-{\bar{K}}$ direction on the surface). The Brillouin zone with the high symmetry points are shown in Fig. 4e. Band structure of CrGeTe$_3$ at 21.2 eV in Fig. 4a,b shows the prominent contributions due to the hybridized Cr 3*d*, Ge 4*p* and Te 5*p* states at RT and LT, respectively. While electronic bands recorded using h$\nu = 84$ eV in Fig. 4c,d at RT and LT showed the prominent contribution only due to the Cr 3*d* states. Broad Cr 3*d* band lies around − 0.5 eV to − 0.7 eV BE at RT in Fig. 4a,c while the band at − 0.2 eV is the Te 5*p* band hybridized with the Cr 3*d* band near $E_F$ (Fig. 4a,b). Low lying VB around $\Gamma$ point consists mainly of Te 5*p* orbitals is also reported in the Ref.^32^. We find that the Te 5*p* band is almost localized at RT (Fig. 4a) indicating polaronic-like states^49^ in the PM phase which lies inside the band gap due to the localized nature of Cr 3*d* bands. Te 5*p* band shows a drastic variation at LT with the hole-like character near $\Gamma$ point (Fig. 4b). Similar hole-like Te 5*p* band and the flat Cr 3*d* band near $E_F$ have been reported in Refs.^31^ and^32^. The other interesting observation is that the bands appearing between -2 eV to -5 eV BE are found to be more hybridized and shifted towards higher BE at LT. Increase in hybridization at LT is due to the small increase in the $U_{eff}$ which gives rise to the exchange interaction between the local moments in Cr atoms through the spin-polarization of the Te 5*p* conduction electrons. Similar broadening of the valence states are also reported in Refs.^23^ and^34^ below the FM transition and has been attributed due to the interplay between localized and itinerant states in this system. However, in the present studies we have clearly shown the contribution of localized and itinerant states across the magnetic phase transition and their role in the magneto-strain effects. The Te 5*p* and Ge 4*p* hybridized bands are not so prominent at 84 eV due to the less photoionization cross-section at this energy. We find that the Cr 3*d* band near $E_F$ at 84 eV in Fig. 4c shows a detwinning effect (marked by arrow) at RT, while at LT in Fig. 4d it not only shows a twinning effect (marked by arrow) but also the Cr 3*d* band found to shift towards higher BE.

**Figure 5:**
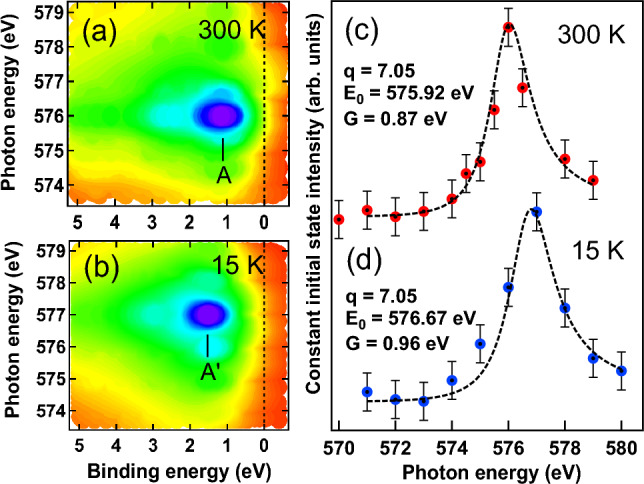
RPES data of CrGeTe$_3$ across Cr $2p-3d$ resonance measured at (**a**) 300 K (RT) and (**b**) 15 K (LT). The constant initial state spectra for feature A and A′ as shown in (**a**) and (**b**) are plotted in (**c**) and (**d**). Solid lines in (**c**) and (**d**) are the fitted Fano line shape (discussed in detail in the text) with Fano parameters *q*, $E_0$ and *G* listed in the figure.

Twinning effect is associated with the crystal structure change that arises at the 2D surface due to the strong interaction between SOC and orbital-lattice coupling. The mechanism of the twinning effect is explained later in the text. Twinning effect has been reported in ARPES data of LaFeAsO^53^ as a function of temperature and tensile strain which has been associated with the momentum dependent splitting of the *d* bands. Similar splitting in Cr 3*d* bands observed in Figs. 4c,d due to the temperature dependent compressive strain. Moreover twinned domains reported in h-BN/graphite system^54^ is associated with the weak vdW interactions. We find that interlayer interactions in CrGeTe$_3$ plays an important role in the twinning effect. The band structure calculation in Figs. 4f,g shows a good matching of bands observed with both 21.2 eV and 84 eV. For instance the band crossing below -2 eV BE in the calculations (Figs. 4f,g) for $U_{eff}$ = 0 and $U_{eff}$ = 1 are prominently visible in 84 eV spectra (Fig. 4c,d) while the hybridized Cr 3*d*, Ge 4*p* and Te 5*p* bands are visible in the 21.2 eV spectra (Fig. 4a,b). There are small differences between the experiment and the theoretical band structure like the shape of the band near $E_F$ for $U_{eff}$ = 0 eV compared with RT band structure (Figs. 4a,f) which is related to the fact that the DFT is a ground-state calculation at 0 K. Moreover, it does not take into account the sample related effects such as the thermal fluctuations, presence of antisite disorders and defects *etc*.

**Figure 6:**
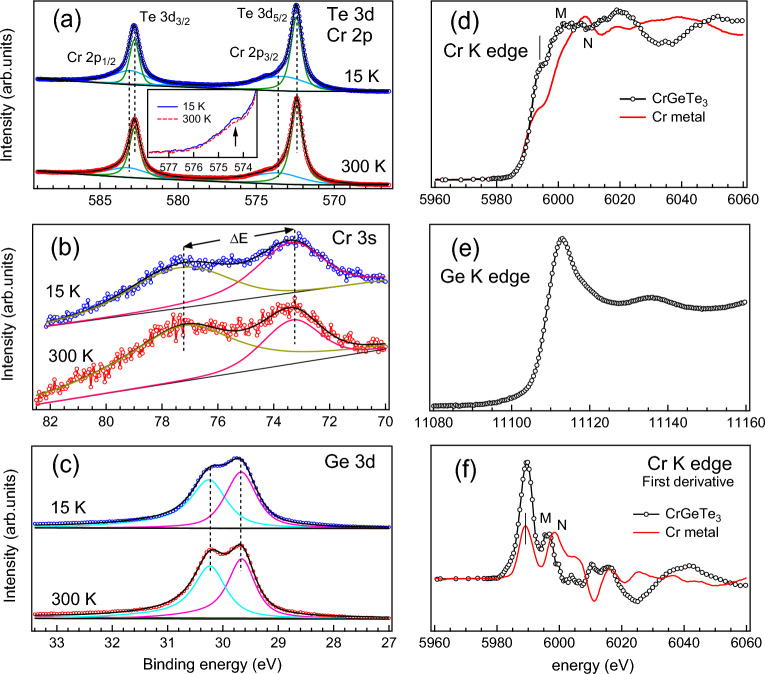
XPS core level spectra at 300 K (RT) and 15 K (LT) showing (**a**) Cr 2p Te 3d, (**b**) Cr 3s and (**c**) Ge 3d levels. Solid lines in (**a**), (**b**) and (**c**) are fitting to the experimental data. Inset in (**a**) shows the magnified Cr 2p$_{3/2}$ region for the changes in the spectral weight at RT and LT. XANES spectra at the K-edge of (**d**) CrGeTe$_3$ and Cr metal and (**e**) Ge. First derivatives of XANES spectra of CrGeTe$_3$ and Cr metal are shown in (**f**).

To understand the nature of Cr 3*d* states in CrGeTe$_3$, we have shown the resonance photoemission (RPES) across Cr 2p-3d transition in Figs. 5a,b. On comparing the contour plots at RT and LT we find that there is not only a shift of the Cr 3*d* resonance feature towards higher BE at LT (see feature A and A′) but also there is increase in the resonance photon energy. We have adopted the standard procedure for the RPES data analysis previously reported by us in Refs.^43,49,50^. The constant initial state (CIS) intensity of the Cr 3*d* features A and A′ at RT and LT are shown in Figs. 5c,d, respectively. The CIS intensity plots are obtained from Figs. 5a,b by plotting the normalized intensity at constant BE position of A and A′. The solid lines in Figs. 5c,d are the fitted Fano line shapes of the form $\frac{(q+\epsilon )^2}{1+\epsilon ^2}$ where $\epsilon =(h\nu -E_0)/G$^55^. Fano parameters *q*, $E_0$ and *G* are listed in Figs. 5c,d at RT and LT. The parameter *q* which gives the information about the discrete/continuum mixing strength found to be the same at both RT and LT. The larger value of $q \approx$ 7.05 indicates that the Cr 3*d* states have strongly localized character. The resonance energy $E_0$ and the half-width of the Fano line *G* gives the information about the hybridization present in the system. Both the increase in the value of $E_0$ and *G* indicates increase in hybridization at LT. Increase in the width is also evident in the Fig. 5b where we can see a small enhancement in the intensity at around 576 eV (light blue region) which indicates strong hybridization of the Cr 3*d* states with the Te 5*p* and Ge 4*p* states and corroborates with the theoretical calculations (see Fig. 3b–e).

In Figs. 6a–c we have shown the changes in the X-ray photoelectron spectroscopy (XPS) core-levels at RT and LT recorded using 750 eV excitation energy. The energy position of Cr 2*p* and Te 3*d* core-levels in Fig. 6a are determined by fitting using the standard procedure^50,51^. We find that there is no change in the spin-orbit splitting for both Cr 2*p* and Te 3*d* at RT and LT but there is a prominent increase in the spectral weight of Cr 2*p* at LT (see inset in Fig. 6a). The area under the Cr 2*p* peak is about 1.6 times higher in LT than in RT. The changes in the spectral weight and line shape in the Cr 2*p* core level observed within our experimental resolution are associated with both the hybridization and correlation present in the valence states. In Fig. 6b Cr 3*s* core level shows the same exchange spitting $\Delta$E$\approx$ 3.98 eV at both RT and LT. Similar $\Delta$E$\approx$ 4 eV has also been observed in CrSi^50^. The contribution of spin-down states (at 73.25 eV) is found to be higher than the spin-up states (at 73.25 eV) LT in Fig. 6b while the opposite has been observed at RT with spin-up states having larger contributions than the spin-down states which indicates that there are changes in the spin DOS with the magnetic transition. Ge 3*d* core level in Fig. 6c shows an unusual broadening of the spin-orbit split peaks. The spin-orbit splitting of the Ge 3*d* peak is $\approx$ 0.55 eV remains the same at both RT and LT but the similar intensity variation as observed in Cr 3*s* peaks in Fig. 6b is also observed in Gd 3*d* peaks. Broadening of the Ge 3*d* peaks is related to the increased lifetime broadening at LT which indicates that more density of valence electrons screen the core hole that leads to the topological transition in this 2D system^56^. Increased bandwidth due to increase in lifetime of Cr $t_{2g}$ state has been reported in CrGeTe$_3$^23^ but our results clearly showed that the bandwidth broadening is mainly governed by the Ge $4s-4p$ electrons which are hybridized with the Cr 3*d* electrons.

**Figure 7:**
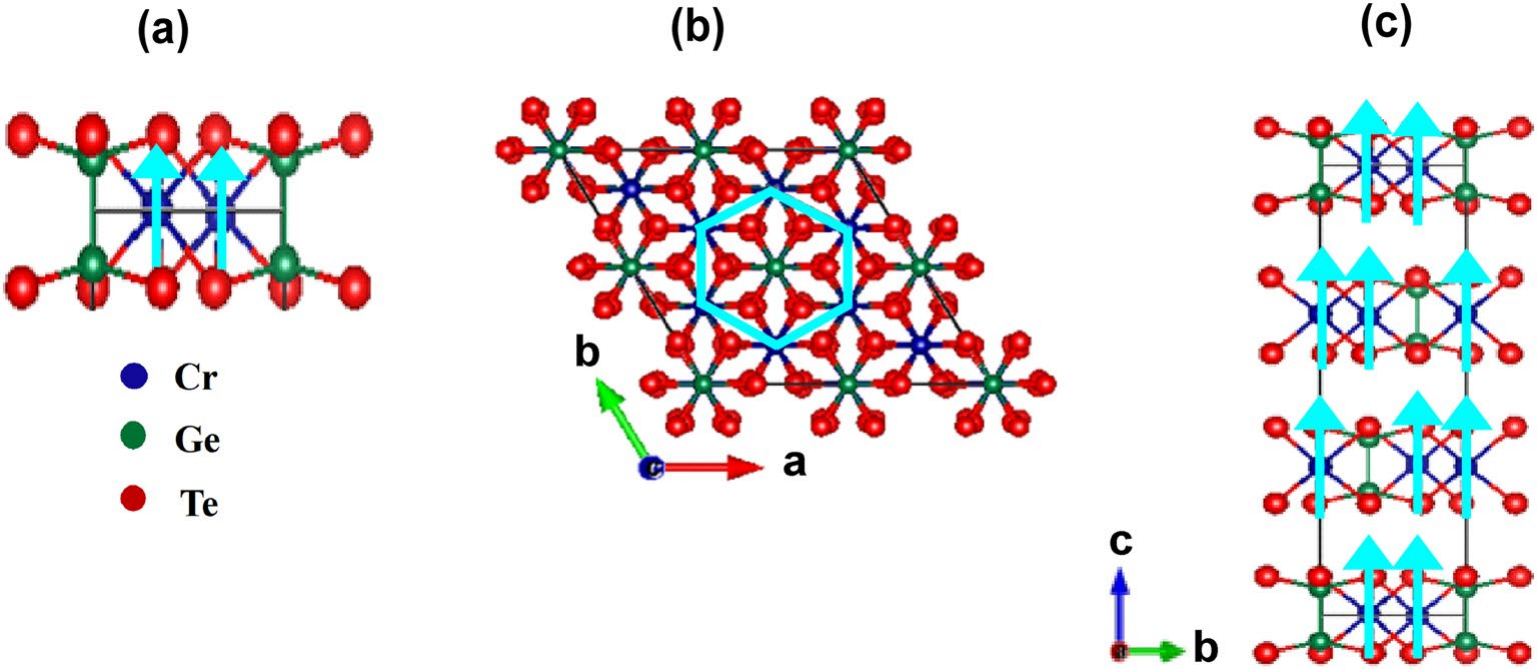
Crystal structure of CrGeTe$_3$ (**a**) single layer (**b**) along ab-plane (in-plane) (**c**) along c-axis (out-of-plane). The arrows show the direction of spins on Cr atoms in (**a**) and (**c**). Hexagonal lattice is shown in ab-plane in (**b**).

Unoccupied electronic structure of CrGeTe$_3$ studied using the X-ray absorption near edge spectroscopy (XANES) measurement at the Cr K-edge and Ge L-edge are shown in Figs. 6d,e. Cr K-edge of CrGeTe$_3$ is compared with the Cr metal. Information about the unoccupied DOS of Cr states can be extracted from the first derivative of the XANES spectra in Fig. 6f. We have observed two important differences between the XANES spectra of CrGeTe$_3$ and Cr metal: (1) shift in the main peak (from $\sim$5998.3 eV for Cr metal to $\sim$5996 eV for CrGeTe$_3$) corresponding to the transition from 1*s* to 4*p* levels (marked by *M* and *N* in Fig. 6d,f) and (2) enhanced pre-edge absorption region in the energy range between 5976 to 5994.5 eV for CrGeTe$_3$ than for Cr metal. The shift of the 1*s* to 4*p* transition to lower energies may be due to the lower Cr–Cr bonding in this system than the Cr metal as reported in Ref.^19^. The lower bonding between Cr 3*d* states leads to more localized 3*d* states in CrGeTe$_3$ than the pure Cr metal and predicts strong hybridization between Cr 4*s* states with Te 5*p* states. Both the Ge K-edge and the Cr K-edge of our sample shows a good matching with the XANES spectra reported in Ref.^19^. Pre-edge feature which corresponds to 1*s* to 3*d* transition showed enhanced intensity for CrGeTe$_3$ in Fig. 6d indicates higher spin-polarized electronic states than the Cr metal which corroborates with our XPS and RPES results. Thus, the observations in XANES and XPS support the presence of more localized Cr states in CrGeTe$_3$ than in Cr metal. However, the microscopic phenomena depends on the Cr 3*d* states which are hybridized with Te 5*p* and Ge $4s-4p$ states.

Hence, the signature of magneto-strain effect in CrGeTe$_3$ from XRD data (Figs. 1d,e) showed an isostructural first-order type transition across $T_C$ with in-plane contraction more than out-of-plane. The out-of-plane magnetization in CrGeTe$_3$ is found to be larger than the in-plane magnetization in the FM phase (Fig. 2a). Magneto-strain effects in the electronic structure showed three important phenomena in the FM phase: (1) shift of bands away from $E_F$, (2) the band broadening and (3) the twinned bands. The mechanism of magneto-strain effects in the electronic bands of CrGeTe$_3$ depends on the changes in the crystal structure which can be explained as follows: the crystal structure of a single layered CrGeTe$_3$ is shown in Fig. 7a where the sequence of atoms in the vdW unit is Te–Ge–Cr–Ge–Te. The vdW gap is along the c-axis between the adjacent units as shown in Fig. 7c. Magnetization result showed that Cr atoms carry the local moment and c-axis is the easy axis of magnetization. The strong SOC defines the spin axis and the direction of magnetization. In the PM phase Cr atoms with local moments are well separated and hence the in-plane Cr–Cr interaction is absent (Fig. 7b). The in-plane Te 5*p* states have weaker hybridization with the Cr 3*d* states (Fig. 7a) and mostly contribute to the carrier density with the polaronic character. While out-of-plane (Fig. 7c) the Cr 3*d* states are hybridized with the near neighbour Ge states and $d-p$ hybridization between Cr 3*d* and Ge 4*p* states governs the semiconducting behavior. Lack of Cr–Cr interaction both in-plane and out of plane gives rise to the detwinned bands in the PM phase. Across the $T_C,$ CrGeTe_3_ undergoes a substantial volume collapse which is a rare phenomenon. Lattice contraction is observed more in-plane than out-of-plane which gives rise to increased interaction between the Cr–Cr, Cr–Ge and Cr–Te atoms. The increase in $U_{eff}$ energy of Cr atom observed in the LT FM phase is because of the in-plane contraction (Fig. 7a,b). This leads to the electron-electron repulsion between the parallel spin orientation of the in-plane Cr atoms and causes a shift of the Cr 3*d* band away from $E_F$. The in-plane spin-polarization is governed by the Te 5*p* states which are hybridized with the Cr 3*d* states (Fig. 7a). The increased hybridization between Cr 3*d* and Ge 4*p* states at LT in the out-of-plane direction gives rise to the broadening of the bands. In Fig. 7c, we can also see that some of the Cr atoms are not arranged one above the other along the c-axis. Hence, the lattice contraction along the out-of-plane direction gives rise to increase interlayer interaction between the Cr atoms which leads to the twinned band structure in this system. Hence, the spin orientation of Cr atoms along c-axis (out-of-plane) depends on the interplay between in-plane $U_{eff}$ and out-of-plane SOC. So, the magneto-strain properties are governed by the out-of-plane interlayer interactions present in this system. The increased spin-polarization of the Te 5*p* states is due to increased hybridization with the in-plane Cr 3*d* states at LT that gives rise to the 2D ferromagnetism in CrGeTe$_3$ and promises it to be a potential candidate for strain engineering and spintronic applications.

## Conclusion

The magnetic interaction in CrGeTe$_3$ is found to be greatly influenced by the microscopic properties like the energy and spatial distribution of electronic DOS. Magnetic transition is found to be strain assisted which leads to the isostructural transition of first order type nature. Larger magnetocrystalline anisotropy is observed in-plane than out of plane. Shift of bands away from $E_F$, band broadening and twinned bands are the signatures of the magneto-strain effects in the electronic bands. Shift of the bands away from $E_F$ is associated with the slight increase in the $U_{eff}$ on Cr 3*d* electrons from $U_{eff}=~0$ to 1 eV across the FM transition and attributed due to in-plane lattice contraction. The SOC between the Cr and Te atoms are found to be much stronger out-of-plane which give rise to detwinned and twinned bands in the PM and FM phases, respectively and associated with the interlayer interactions. Broadening of the VB and the core-levels at LT are associated with the hybridization between Ge and Cr orbitals which govern the semiconducting behavior in this system. Hence, the magneto-strain effects in CrGeTe$_3$ results in increased in-plane $U_{eff}$ and out-of-plane SOC. The interplay between $U_{eff}$ and SOC gives rise to 2D ferromagnetism in CrGeTe$_3$. Tunable vdW gap with temperature and magnetic field promises CrGeTe$_3$ for novel spintronics and straintronic applications.

## Methods

### Sample preparation and characterization

High quality CrGeTe$_3$ crystals were grown using self-flux technique. High purity Cr (99.995%, Alfa Aesar), Ge (99.999%, Alfa Aesar) and Te (99.999%, Alfa Aesar) were taken as starting materials with a molar ratio of 1:3:18 respectively. The precursors mixed and sealed in an evacuated quartz tube, another end of the quartz tube was filled with quartz wool as the medium for centrifugation. Sealed quartz tube was placed in a furnace and heated to 700 $^{\circ }$C for 10 h, and cooled down to 480 $^{\circ }$C with a cooling rate of 6 $^{\circ }$C/h. At this temperature quartz tube was taken out from the furnace and flipped over gently and centrifuged for 30 sec in order to obtain high quality CrGeTe$_3$ single crystals. The final composition after sample preparation has been determined by EDAX attached to a scanning electron microscope Carl Zeiss FESEM (model Sigma 002).

### Crystal structure studies

X-ray diffraction (XRD) measurements have been performed on both the single crystal as well as on the powder sample of CrGeTe$_3$. Small pieces of single crystals are grounded into very fine powder in mortar and pestle for 2 hours. Lab based XRD was performed using a commercial X-ray diffractometer from Bruker model D8 Advance with Cu K$_\alpha$ source. Detailed temperature dependent synchrotron XRD measurements using 15 keV excitation energy were performed at the angle-dispersive X-ray diffraction (ADXRD) beamline BL-12 at Indus-2 synchrotron radiation source. Liquid Helium based flow type cryostat with the temperature stability of 0.15 K is used to perform temperature dependent XRD measurements. Si(111) based double crystal monochromator is used to achieve high spectral resolution of 1 eV at 10 keV. Synchrotron XRD patterns were recorded on powder samples using Image plate Mar-345 detector. The sample to detector distance and photon energy were calibrated using LaB$_6$ NIST standard. The accuracy of determining the peak position of LaB$_6$ NIST standard sample at 15 K is ± 0.005 deg. The XRD patterns are generated from the diffraction rings obtained by Image plate data using Fit2D software. XRD analysis for Le Bail refinement was carried out using the JANA2000 package^57^.

### Magnetic studies

Temperature dependent magnetization M(T) at 0.1 T and 5 T magnetic field (H) performed along H//ab and H//c using 16 Tesla vibrating sample magnetometer (Dynacool, M/s. Quantum Design, USA) in zero-field-cooled (ZFC), Field-cooled-cooling (FCC) and Field-cooled-warming (FCW) protocols. FCW data overlay with the FCC data, hence it is not shown here. Field dependent magnetization M versus H upto 8 T performed in PM phase (at 300 K), near short range ordering (at 200 K) and FM phase (at 4 K).

### Electronic structure studies

Temperature dependent photoemission measurements were performed at the undulator based Angle Resolved Photoelectron Spectroscopy beamline (ARPES BL-10), Indus-2 using Phoibos 150 electron energy analyser. The single crystal was cleaved in the vacuum of $\sim 5~\times 10^{-11}$ mbar to get atomically clean surface. All the photoemission measurements were carried out in the base vacuum of $\sim 7~\times 10^{-11}$ mbar in the analysis chamber of ARPES BL-10. RPES measurements carried out by recording the VB spectra at the photon excitation energies ranging from 570 to 580 eV across the Cr $2p-3d$ resonance with the energy resolution of 0.26 eV. High resolution ARPES measurements were carried out at  15 K (LT) and 300 K (RT) using synchrotron radiation h$\nu = 84$ eV with photon flux $\approx 10^{11}$ photons/sec and He-1 source h$\nu = 21.2$ eV with photon flux $\approx 10^{16}$ photons/s. The energy resolution at 84 eV is 40 meV and 21.2 eV is 20 meV with 0.2$^o$ angular resolution. Synchrotron XPS core level spectra were recorded at h$\nu = 750$ eV with 0.3 eV energy resolution. The BE scale was calibrated with the Ag 3*d* lines and Ag Fermi edge following the standard procedure^58,59^. Charging effect has been taken care by measuring the data at lowest synchrotron photon flux. We have neither seen the distorted spectra nor the energy shift while measurement. Moreover the data collected at different times showed the same intensity which confirms that there is no charging effect. Similar procedure to avoid the charging effect has been reported in Ref.^34^. All the core-level peaks are fitted using a least-square error minimization routine with Doniac-Sunjic line shape^60^. The inelastic background has been subtracted using the Tougaard method^61^. The instrumental broadening is considered by convoluting the line shapes with a Voigt function. The instrumental parameters have been kept fixed during the fitting. XANES measurements were performed at the EXAFS beamline BL-9 at Indus-2 synchrotron radiation source. XANES measurement was performed in fluorescence mode at Cr K-edge in the energy range from 5960 to 6060 eV and Ge K-edge in the energy range from 11080 to 11160 eV.

### Density functional theory

First-principles spin-polarized calculations within the DFT were performed using the generalized gradient approximation (GGA) for exchange and correlation potential^62^. Vienna ab-initio simulation package (VASP)^63^, was used which solves the Kohn-Sham equations using a plane wave expansion for the valence electron density and wave functions. The projector augmented wave (PAW)^64,65^ potential describes the interactions between the ions and electrons. The PAW potential used in the calculation treats ${ 3d4s4p}$ as valence states for Cr and Ge and ${ 5s5p}$ as valence states for Te. The expansion of electronic wave functions in plane waves was set to a kinetic energy cut-off ($E_{cutoff}$) of 350 eV. The Brillouin-zone was sampled using Monkhorst-Pack k-point mesh^66^ of 8 $\times$ 8 $\times$ 8 (128 k-points in the irreducible Brillouin zone (IBZ)) for the bulk structure. The energy optimization was carried out with respect to a k-point mesh and $E_{cutoff}$ to ensure convergence of the total energy to within a precision better than 1 meV/atom. The structural relaxations were performed using the conjugate gradient algorithm until the residual forces on the atom were less than 0.01 eV/$\text{\AA }$ and stresses in the equilibrium geometry were less than 5$\times$10$^{-2}$ GPa. The tetrahedron method with Bl$\ddot{o}$chl corrections^64,65^ was used to perform the total electronic energy and DOS calculations. To account for the Coulomb correlation interaction within the Cr 3*d* shell, we additionally considered the PBE XC potential corrected according to GGA+ U method using the simplified Dudarev approach^67^ including the SOC.

## Supplementary Information

Supplementary Information.

## Data Availability

The datasets used and/or analysed during the current study available from the corresponding author on reasonable request.
