# Supplementary material for: Magneto-strain effects in 2D ferromagnetic van der Waal material CrGeTe3
Source: Sci Rep. 2023 May 26;13:8579. doi: 10.1038/s41598-023-35038-2 (PMC10219987; doi:10.1038/s41598-023-35038-2)
Supplement: Supplementary file 1 — Supplementary Information. [file 41598_2023_35038_MOESM1_ESM.pdf]

Supplementary Information

Magneto-strain effects in 2D ferromagnetic van der Waal material $CrGeTe_3$

Kritika Vijay[1,2], Durga Sankar Vavilapalli[3], A. Arya[4], S. K. Srivastava[1], Rashmi Singh[5], Archna Sagdeo[1,2], S. N. Jha[2,6], Kranti Kumar[7], and Soma Banik[1,2]*

[1]*Accelerator Physics and Synchrotrons Utilization Division, Raja Ramanna Centre for Advanced Technology, Indore, 452013, India.*

[2]*Homi Bhabha National Institute, Training School Complex, Anushakti Nagar, Mumbai, 400094, India.*

[3]*Materials Design Division, Department of Physics, Chemistry and Biology (IFM), Linköping University, 581 83, Linköping, Sweden.*

[4]*Glass and Advanced Materials Division, Bhabha Atomic Research Centre, Mumbai, 400085, India*

[5]*Laser Materials Development & Devices Division, Raja Ramanna Centre for Advanced Technology, Indore,452013, India.*

[6]*Beamline Development and Application Section, Bhabha Atomic Research Centre, Mumbai, 400085, India*

[7]*UGC-DAE Consortium for Scientific Research, Khandwa Road, Indore 452001, India.*

***Email: soma@rrcat.gov.in**

Probing crystal structure of $CrGeTe_3$ using X-ray diffraction:

The XRD pattern of $CrGeTe_3$ single crystal recorded with the Cu K_α source is shown in Supplementary Figure 1. The Le bail refinement of the single crystal was performed using hexagonal R-3:H space group and also shown in Supplementary Figure 1. The refined lattice parameters obtained are a= 6.8576 Å and c= 20.5979 Å also mentioned in the text of the manuscript.

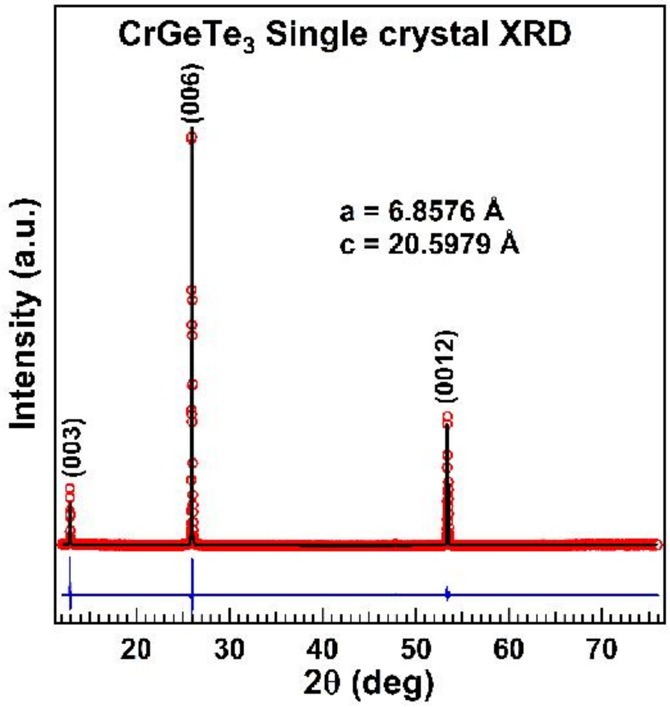

Supplementary Figure 1: Le Bail refinement of $CrGeTe_3$ single crystal showing the peaks indexed with the R-3:H space group. Open red circle shows the experimental data recorded with Cu K_α source, solid black line shows the calculated data and the blue line is the difference calculated by subtracting the experimental data and calculated data.

After grinding the single crystal for about 2 hours in mortar and pestle, we have again collected the XRD pattern of $CrGeTe_3$ powder with the Cu K_α source as shown in Supplementary Figure 2. We find that there are some additional phases (marked by arrows in Supplementary Figure 2) which may have occurred due to the developed strain while grinding. We find that the Le bail refinement of the powder sample performed using the single hexagonal R-3:H structure in Supplementary Figure 2 does not fit the whole XRD pattern. Hence, there is a possibility of phase segregation in $CrGeTe_3$ which has not been explored in detail till date. $CrGeTe_3$ is a well-known phase change material and both crystalline to crystalline and crystalline to amorphous phase changes have been reported with the applied pressure [1].

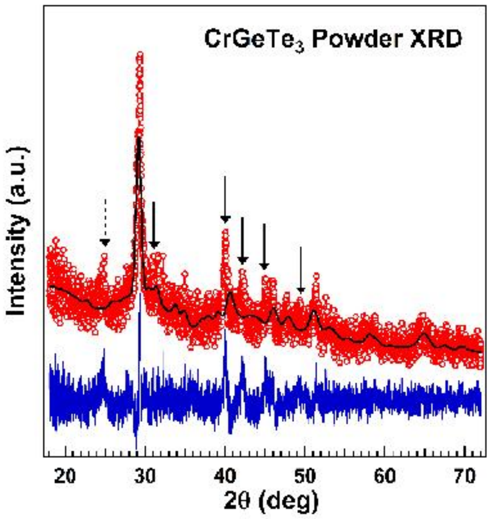

Supplementary Figure 2: XRD pattern of $CrGeTe_3$ powder after grinding showing the peaks fitted with R-3:H space group. Open red circle shows the experimental data recorded with Cu K_{α} source, solid black line shows the calculated data with R-3:H space group and the blue line is the difference calculated by subtracting the experimental data and calculated data.

To better understand the phase segregation in $CrGeTe_3$ we have performed the synchrotron XRD (SR-XRD) measurements at 15 keV. The SR-XRD data in Supplementary Figure 3 looks better than the Cu K_{α} data in Supplementary Figure 2 and the phase segregation has been clearly visible. The calculated XRD pattern with R-3:H space group is shown in Supplementary Figure 3. Additional phase segregated peaks are marked by an arrow in the figure.

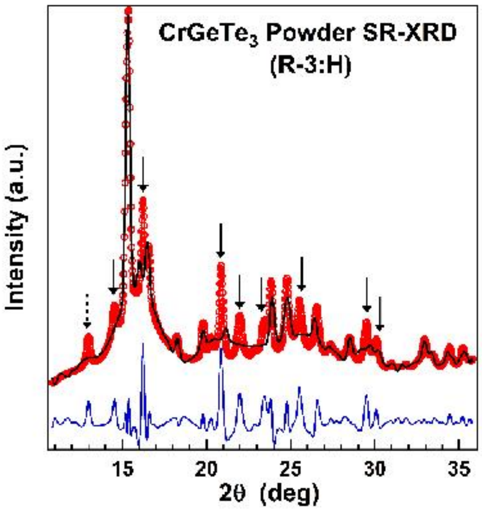

Supplementary Figure 3: SR-XRD pattern of $CrGeTe_3$ powder showing the peaks fitted with R-3:H space group and the phase segregated peaks marked by arrows. Open red circle shows the experimental data, solid black line shows the calculated data with R-3:H space group and the blue line is the difference calculated by subtracting the experimental data and calculated data.

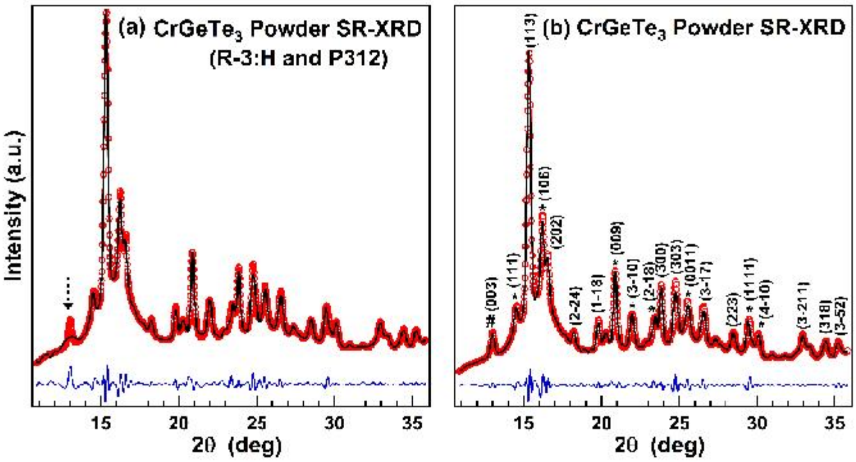

Supplementary Figure 4: SR-XRD pattern of $CrGeTe_3$ powder showing the peaks fitted with R-3:H + P312 space groups in (a) and R-3:H + P312 + R-3m:H space groups in (b). Open red circle shows the experimental data, solid black line shows the calculated data and the blue line is the difference calculated by subtracting the experimental data and calculated data.

In SR-XRD we find a prominent low symmetry non-centrosymmetric P312 structure along with the centrosymmetric R-3:H structure of $CrGeTe_3$ as shown in Supplementary Figure 4 (a). P312 structure is well reported in Ref. [2] which has been observed in XRD. Moreover the presence of both the crystalline and amorphous phases with same local structure has been reported from EXAFS in Ref. [3]. In Supplementary Figure 4(a) we could able to fit most of the peaks considering R-3:H and P312 structure of $CrGeTe_3$ however, a peak at lower angle marked by dashed arrow in Supplementary Figure 4(a) could not be fitted with both the phase so there is a possibility of one more additional phase which may arise due to the clustering of Ge and Te in this system or the eutectic phase and fitted with GeTe type R-3m:H structure as shown in Supplementary Figure 4(b). The asterisk (*) marked peaks are due to the additional contribution of P312 structure while the hash (#) marked peak corresponds to the GeTe type eutectic phase in $CrGeTe_3$. The difference between the calculated data and the experimental data in Supplementary Figure 4(b) (see blue line) shows a good fitting with all the phases.

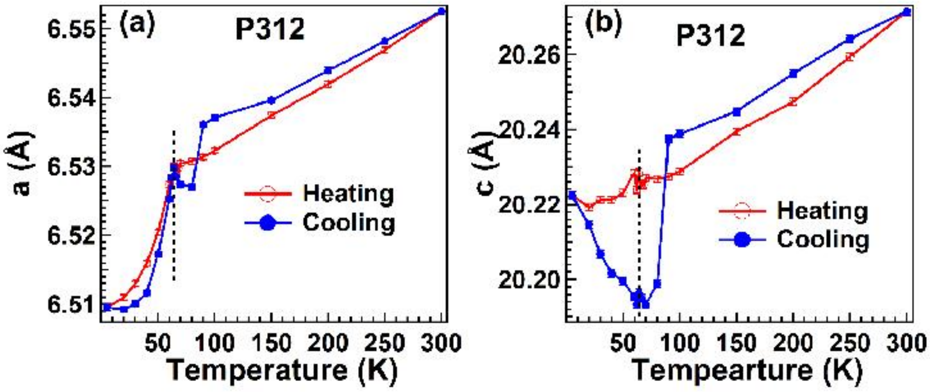

Supplementary Figure 5: Lattice parameter a and c variation with temperature for the P312 phase of $CrGeTe_3$.

The lattice parameter variation for the dominating R-3:H phase is discussed in the manuscript while the P312 phase is shown in Supplementary Figure 5(a) and (b). We can see that the lattice parameter *a* does not show much differences in heating and cooling while the most differences are observed in the lattice parameter *c*. This is expected as the sudden jump in the lattice parameter and the hysteresis indicates the magnetic interaction between the Cr atoms and the presence of magneto-strain effects in this system.

The changes in the lattice parameters for the GeTe phase are shown in Supplementary Figure 6(a) and (b). We can see very clearly that there is decrease in the lattice parameters with temperature for both heating and cooling cycles. GeTe has ferroelectric phase transition around ≈650 K-700 K [4,5] which lies much above the measured temperature range. Hence, the GeTe phase does not contribute to the magneto-strain effects present in this system.

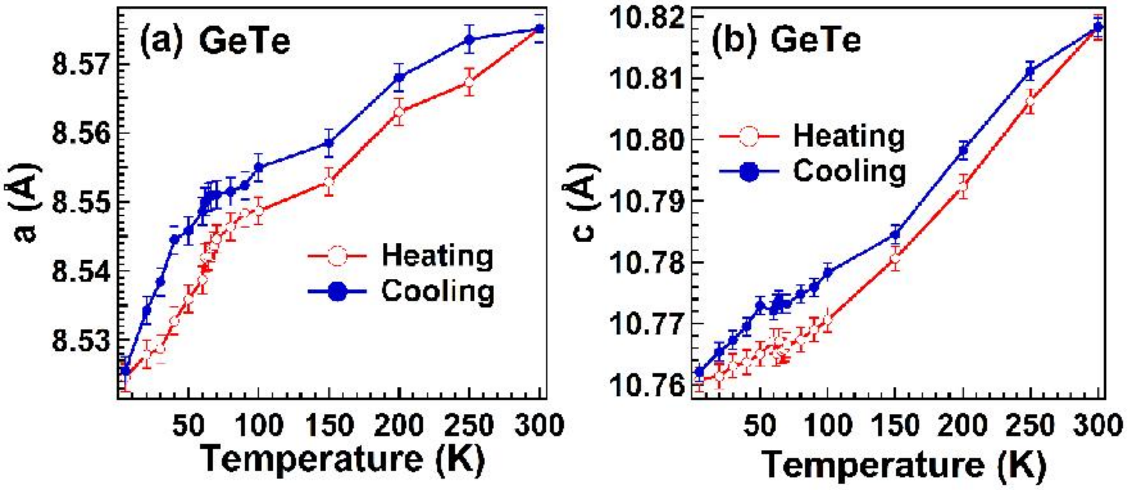

Supplementary Figure 6: Lattice parameter a and c variation with temperature for the R-3m:H phase of GeTe.

We have determined the contribution of magneto-strain effects in the crystal structure. The detailed procedure is described in Ref. [6]. The residual micro-strain (ε) which is associated with the magneto-strain effects can be calculated using the formula $\varepsilon = \Delta d/d$, where d is the peak position and $\Delta d = (\Delta d_{observed}^2 - \Delta d_{instrumental}^2)^{1/2}$ is the width of the peak after subtracting the instrumental broadening. The instrumental broadening has been obtained from the LaB_6 NIST standard sample. The error in estimating $\Delta d_{instrumental}$ is about ≈0.0003 Å. The value of ε has been calculated as a function of temperature in the heating cycle for the 15.35 deg peak with d= 3.069 Å and is shown in the Fig. 7. The value of strain is found to be quite large in this van der Waal system as compared to other metallic systems [6]. We can clearly see that the ε at 300 K (14.313 $\times 10^{-2}$) is smaller than at 5 K (14.413 $\times 10^{-2}$). The error in the estimation of strain is $\approx 10^{-5}$. Similar error in the strain estimation is reported in Ref. [7]. The increase in the value of ε at

lower temperatures clearly shows the contribution due to the magneto-strain effects which enhances at lower temperature and depends on the magnetic interactions present in $CrGeTe_3$. The contraction in the lattice parameters as a function of temperature indicates a compressive strain in the system. The estimated temperature induced compressive strain is ≈0.7%.

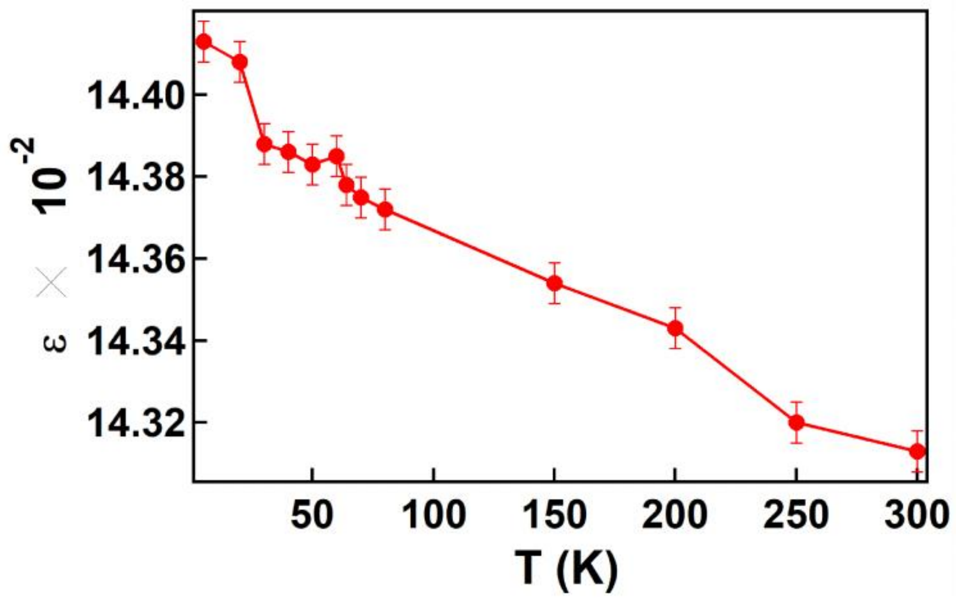

Supplementary Figure 7: Estimated ε as a function of temperature for $CrGeTe_3$.

Magnetization studies:

Supplementary Figure 8 shows the magnetization measurements in zero-field-cooled (ZFC) and field-cooled-cooling (FCC) protocols in the temperature range between 105 to 300 K, at H = 0.1 T (left scale) and 5 T (right scale). ZFC and FCC data are shown by solid and dashed lines, respectively. The magnetic field was applied parallel to the ab plane and along the c-axis. The magnetization curve shows prominent short range ferromagnetic ordering at 0.1 T around 220 K (marked by vertical arrow). At 5 T field this short range ordering is hardly visible. The reason for the suppression of the short range ordering at 5 T field is because the applied magnetic field tries to align the spins in the direction of the field.

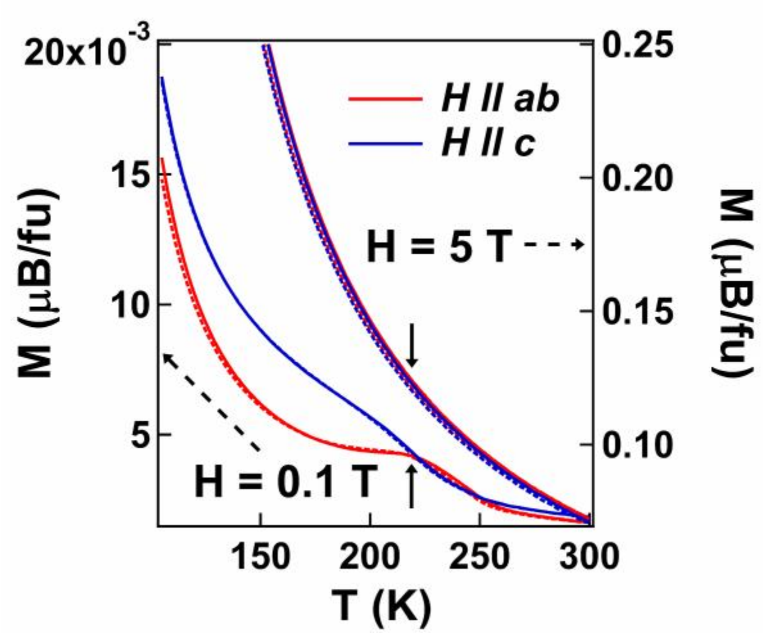

Supplementary Figure 8: The region between 105 to 300 K of M versus T data at 5 T and 0.1 T are shown. The signature of short range ordering is marked by vertical arrows in H//ab plane and H//c axis at 5 T and 0.1 T magnetic field.

References:

[1] Yu, Z., *et al.*, Pressure-Induced Structural Phase Transition and a Special Amorphization Phase of Two-Dimensional Ferromagnetic Semiconductor $Cr_2Ge_2Te_6$, J. *Phys. Chem. C* **123**, 13885 (2019).

[2] Carteaux, V., Brunet, D., Ouvrard, G., & Andre, G., Crystallographic, magnetic and electronic structures of a new layered ferromagnetic compound $Cr_2Ge_2Te_6$, *J. Phys.: Condens. Matter* **7**, 69 (1995).

[3] Hatayama, S., *et al.*, Cr-Triggered Local Structural Change in $Cr_2Ge_2Te_6$ Phase Change Material, *ACS Appl. Mater. Interfaces* **11**, 43320 (2019).

[4] Zhu, C., *et al.*, Enhanced Thermoelectric Performance of GeTe-Based Composites Incorporated with Fe Nanoparticles, ACS Appl. Mater. Interfaces 14, 38854 (2022).

[5] Liu, W. –Di., *et al.*, High-Performance GeTe-Based Thermoelectrics: from Materials to Devices, Adv. Energy Mater. 10, 2000367 (2020).

[6] Banik, S., Arya, A., & Sinha, A. K., Direct hybridization gap from intersite and onsite electronic interactions in $CeAg_2Ge_2$, *RSC Adv.* **10**, 24343 (2020).

[7] Polvino, S. M., “Accuracy, Precision, and Resolution in Strain Measurements on Diffraction Instruments”, Thesis, Columbia University (2011); https://core.ac.uk/download/pdf/161437714.pdf
